# A Rare Case of Midostaurin-Associated Sweet's Syndrome

**DOI:** 10.1155/2022/1099005

**Published:** 2022-04-22

**Authors:** Hesham Yasin, Tessa Laytem, Grerk Sutamtewagul, Sabarish Ayyappan

**Affiliations:** University of Iowa Health Care, Iowa City, Iowa, USA

## Abstract

Acute febrile neutrophilic dermatosis which is referred as Sweet's syndrome (SS) is a dermatological condition characterized by fever, erythematous rash, and leukocytosis. SS can be idiopathic or associated with malignancies or medications. We present a rare case of SS which developed shortly after starting midostaurin in a patient with acute myelogenous leukemia (AML).

## 1. Introduction

Acute febrile neutrophilic dermatosis, known as Sweet's syndrome (SS), is a dermatological condition characterized by abrupt appearance of erythematous rash, accompanied frequently by the presence of fever and leukocytosis. Majority of cases are idiopathic or malignancy-associated but rarely can be drug-induced. Midostaurin, a recently FDA-approved agent, has shown improved outcomes in FLT3-mutated acute myeloid leukemia (AML). To date, there are three reported cases in which midostaurin administration had a temporal association with the onset of SS. We are presenting here another similar case.

## 2. Case Presentation

**Figure 1:**
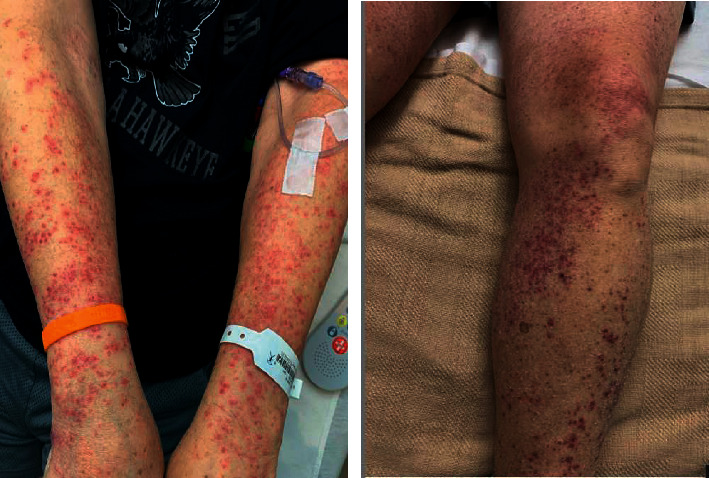
Maculopapular rash in the patient's bilateral upper and lower extremities.

**Figure 2:**
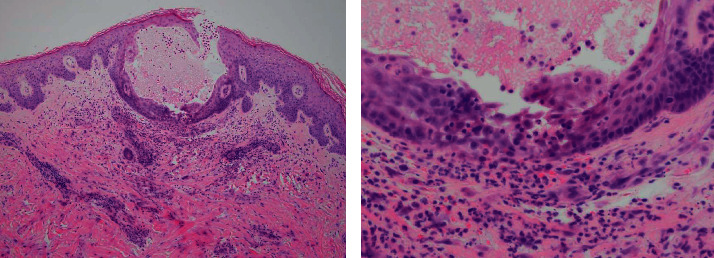
Microscopic view of the pathological specimen from skin biopsy: spongiosis with focal intraepidermal vesiculation as well as papillary dermal edema with upper dermal neutrophilic and lymphocytic inflammation with extravasated erythrocytes.

A 60-year-old Caucasian male with no significant medical history presented to his primary care physician with a one-month history of fatigue, decreased appetite, 15-pound weight loss, and easy bruising and bleeding. Physical exam at that time was remarkable only for moderately pale conjunctiva. Laboratory evaluation was remarkable for a hemoglobin level of 8.3 g/dL, WBC count of 2.6 k/mm^3^, and platelet count of 28 k/mm^3^. The peripheral blood differential was remarkable for occasional blasts and elevated lactate dehydrogenase (LDH) at 402 U/L. The kidney and liver function tests were normal. Given the pancytopenia and presence of blasts in the peripheral smear, a bone marrow aspiration and biopsy were obtained, which showed acute myeloid leukemia (AML) with genetic studies showing mutated TP53, NPM1, PTPN11, and FLT3. The patient was admitted to the hospital and started on induction chemotherapy with cytarabine and daunorubicin (7 + 3 regimen). He tolerated the initial course and had no significant events except for expected gradual decrement in blood cell count. On day eight of hospitalization, the patient was started on midostaurin due to positive FLT3 mutation and high-risk disease. Within 12 hours of starting midostaurin (day 9), he developed intermittent fevers. Routine infectious workup was sent, and cefepime was started empirically. The patient's fevers continued, and given the persistent fever, cefepime was discontinued two days later (day 11) and antibiotics were escalated to piperacillin-tazobactam and vancomycin. No source of infection was found at that time. On the same day (day 11), the patient reported a mildly painful and nonpruritic rash. He first noticed it on both arms, and subsequently, the rash spread to bilateral lower extremities. On exam, the patient had bilateral pink-to-erythematous, scattered, edematous papules with very few hemorrhagic changes (Figure 1). There was no other rash seen on his bilateral upper and lower extremities. Blood work at that time was notable for a hemoglobin level of 6.4 g/dL, WBC count of 0.4 k/mm^3^, platelets of 21 k/mm^3^, and neutrophil count of 250/mm^3^. The patient then underwent punch biopsy of a single skin lesion on the upper arm. Results showed findings of spongiosis with focal intraepidermal vesiculation as well as papillary dermal edema with upper dermal neutrophilic and lymphocytic inflammation with extravasated erythrocytes. These findings, in the context of the clinical picture, were consistent with SS (Figure 2). The patient was then started on prednisone 60 mg daily. Over the next few days, the patient demonstrated rapid improvement of the rash. Prednisone tapering was started after seven days with planned incremental decrease by 10 mg every four days until discontinuation. The patient continued to spike fevers for seven days after the prednisone was started. Repeat infectious workup did not localize any source of infection, and therefore, it was presumed the fever was due to SS. Midostaurin was continued after the diagnosis of SS. The patient was continued to follow up with the leukemia clinic on a regular basis. The patient went into remission and then started AML consolidation therapy. To date, he has not had recurrence of the rash. The list of the drugs the patient was taking 30 days prior to the development of rash is presented in Table 1.

## 3. Discussion

Acute febrile neutrophilic dermatitis, first described by Sweet in 1964 and hence named “Sweet's syndrome” after him [1], is an inflammatory dermatological disease caused by extensive infiltration of the dermis and epidermis by neutrophils [2]. It is characterized by fever, neutrophilia, and tender erythematous skin lesions that can be papules, nodules, or plaques [3]. Sweet's syndrome is classified into three subtypes: classical (or idiopathic) SS, malignancy-associated SS, and drug-associated SS [3]. Classical SS is diagnosed when malignancy is ruled out and no temporal association with a drug can be established. It is more common in women and often associated with recent upper respiratory tract or gastrointestinal infection, pregnancy, and inflammatory bowel disease [4–6]. Hematological malignancies account for the largest proportion of malignancy-associated SS, and in a review, AML was the commonest malignancy to be associated with this subtype [7, 8]. There are multiple agents that have been reported to be associated with development of drug-associated SS. Frequently reported medications associated with SS include granulocyte-colony stimulating factor, trimethoprim-sulfamethoxazole, azathioprine, minocycline, and all-trans retinoic acid [9–11]. Fever is the most common extracutaneous symptom which can precede the rash by several days to weeks [3]. Dermatological manifestations are usually painful, red-to-purple-colored papules or nodules which may coalesce into larger plaques. Lesions are usually asymmetrically distributed and commonly occur on the face, upper extremities, and neck [12]. There are several reports of extracutaneous manifestations such as arthralgia and arthritis, encephalitis, and ocular manifestations in the literature [13–15]. Diagnostic criteria are summarized in Table 2. To make a diagnosis, presence of two major criteria and at least two minor criteria is required. To establish the diagnosis of malignancy-associated SS, temporal relationship must occur between malignancy and symptoms in addition to meeting the criteria for diagnosis of classical SS. Diagnostic criteria for drug-induced SS are summarized in Table 3, and all of the five criteria must be present to establish the diagnosis [3, 9]. Histological examination will reveal a characteristic diffuse infiltrate predominantly consisting of mature neutrophils typically located in the upper dermis [2]. Lesions left untreated can persist for weeks to months [16]. Treatment of malignancy or discontinuation of the offending drug can result in subsequent improvement and disappearance of symptoms [3]. Topical or intralesional corticosteroids can be used to treat patients who have a small number of lesions [17]. Systemic corticosteroids are the mainstay for therapy and usually result in dramatic improvement of symptoms within 72 hours. The starting prednisone dose ranges from 0.5 to 1.5 mg/kg/day which is slowly tapered over several weeks [2, 17]. Potassium iodide has been previously reported but can be difficult to titrate and safely administer [18]. There are many reports of steroid-sparing agents including colchicine, dapsone, and cyclosporine [17, 19, 20].

Midostaurin, a tyrosine kinase inhibitor, is a targeted drug therapy for FLT3-mutated AML [21]. The most common side effects for this agent are cytopenia, neutropenic fever, and gastrointestinal and pulmonary complaints. Rash was reported as a side effect, but there are no reports of SS in clinical trials [22]. To the best of our knowledge, there are three case reports of patients who developed Sweet's syndrome while being treated with midostaurin [23–25]. Our patient developed SS just three days after starting the treatment. Among all the listed medications that the patient was taking in the last 30 days (Table 1), none of these medications were reported in the literature to be associated with SS. The patient's underlying AML is very likely to be the causative inducer of SS, but the temporal association with midostaurin administration was assumed to contribute to the pathogenesis. Our patient met the five criteria required for drug-induced SS (Table 3), and the patient had resolution with steroid treatment. In conclusion, midostaurin is an effective agent against FLT3-mutated AML, and it has been increasingly used in clinical practice. It is important to consider midostaurin as a culprit medication in the setting of SS.

## Figures and Tables

**Table 1:** Drug administered to the patient prior to the onset of rash (none has been reported to be associated with Sweet's syndrome except midostaurin).

Medication	Days the medication was administered prior to the onset of rash
Amoxicillin-clavulanic acid	30
Cytarabine	11
Daunorubicin	11
Allopurinol	11
Ciprofloxacin	11
Isavuconazonium	3
Midostaurin	3
Piperacillin-tazobactam	1
Cefepime	2
Vancomycin	1

**Table 2:** Diagnostic criteria for Sweet's syndrome.

Major criteria	Minor criteria
Abrupt onset of painful erythematous papules, nodules, or plaques	Fever >38°C

Histopathological findings of dense neutrophilic infiltrates without evidence of leukocytoclastic vasculitis	Association with hematologic or visceral malignancy, inflammatory disease, or pregnancy, or preceded by upper respiratory tract infection, gastrointestinal infection, or vaccination

	Dramatic response to treatment with systemic steroids or potassium iodide

	Abnormalities in laboratory tests (three of four): erythrocyte sedimentation rate >20 mm/h; high C-reactive protein; leukocytes >8000, with >70% neutrophils

**Table 3:** Diagnostic criteria for drug-induced Sweet's syndrome.

Diagnostic criteria	Presence in our patient
Abrupt onset of painful erythematous papules, nodules, or plaques	Yes
Histopathological findings of dense neutrophilic infiltrates without evidence of leukocytoclastic vasculitis	Yes
Fever >38°C	Yes
Temporal relation between use of medication and clinical presentation or relapse with readministration	Yes
Disappearance of lesions after discontinuation of drug or treatment with systemic steroids	Yes

## Data Availability

The data can be found in the EMR of the University of Iowa Hospital.
